# Patients’ willingness to share digital health and non-health data for research: a cross-sectional study

**DOI:** 10.1186/s12911-019-0886-9

**Published:** 2019-08-08

**Authors:** Emily Seltzer, Jesse Goldshear, Sharath Chandra Guntuku, Dave Grande, David A. Asch, Elissa V. Klinger, Raina M. Merchant

**Affiliations:** 1 Penn Medicine Center for Digital Health, University of Pennsylvania, 3400 Civic Blvd, Philadelphia, PA USA; 2 Department of Emergency Medicine, Perelman School of Medicine, University of Pennsylvania, Philadelphia, PA USA; 3 Department of Computer and Information Science, University of Pennsylvania, Philadelphia, PA USA; 4 Penn Medicine Center for Health Care Innovation, University of Pennsylvania, Philadelphia, PA USA; 5 Division of General Internal Medicine, Perelman School of Medicine, University of Pennsylvania, Philadelphia, USA; 6 The Center for Health Equity Research and Promotion, Michael J Crescenz VA Medical Center, Philadelphia, PA USA

**Keywords:** Data privacy, Data donation, mHealth, Digital health, Social media

## Abstract

**Background:**

Patients generate large amounts of digital data through devices, social media applications, and other online activities. Little is known about patients’ perception of the data they generate online and its relatedness to health, their willingness to share data for research, and their preferences regarding data use.

**Methods:**

Patients at an academic urban emergency department were asked if they would donate any of 19 different types of data to health researchers and were asked about their views on data types’ health relatedness. Factor analysis was used to identify the structure in patients’ perceptions of willingness to share different digital data, and their health relatedness.

**Results:**

Of 595 patients approached 206 agreed to participate, of whom 104 agreed to share at least one types of digital data immediately, and 78% agreed to donate at least one data type after death. EMR, wearable, and Google search histories (80%) had the highest percentage of reported health relatedness. 72% participants wanted to know the results of any analysis of their shared data, and half wanted their healthcare provider to know.

**Conclusion:**

Patients in this study were willing to share a considerable amount of personal digital data with health researchers. They also recognize that digital data from many sources reveal information about their health. This study opens up a discussion around reconsidering US privacy protections for health information to reflect current opinions and to include their relatedness to health.

**Electronic supplementary material:**

The online version of this article (10.1186/s12911-019-0886-9) contains supplementary material, which is available to authorized users.

## Background

In 2012, the retailer Target sent advertisements for baby products to a teen who had not disclosed her pregnancy to her parents. Target had concluded the teen was pregnant after she purchased items like unscented lotion and cotton balls, which figured into algorithms predicting pregnancy [1]. The algorithm was allegedly accurate, but the tracking practices of Target were criticized after it was reported to the public [1, 2]. Individual customers reportedly complained and reported that predicting pregnancy from purchases was “creepy” [1]. After the public response Target was reported to have modified its marketing practices, instead of only sending baby supply coupons to women that their algorithm deemed pregnant, Target would send baby supply coupons with other home goods items mixed in [1]. “As long as a pregnant woman thinks she hasn’t been spied on, she’ll use the coupons” [2]. The sensitive nature of early pregnancy makes the practice of targeted marketing seem particularly invasive. There are many regulations enacted to protect traditional clinical health information, but there is less guidance for how health related digital data should be protected.

Contemporary practices to safeguard the privacy of health related data, such as HIPAA privacy rules, emerged at a time when health data were largely seen as the products of clinical encounters [3]. But health is revealed in a wide range of individual behaviors that occur outside the health care system—in purchases, communications, searches, locations—and an increasing share of those activities are captured electronically where they can be linked and analyzed. These data offer promise to advance research on individual or public health – for instance in uncovering insights on manifestations and sequelae of mental health, hospital encounters, and outbreaks [4]. Public acceptability of using these data for health purposes is, however, unknown and likely dynamic [5]. The promise of applying disparate digital data to health insights sits alongside enormous practical uncertainties about logistics, acceptability, perceived and actual value.

Prior work suggests that many individuals are willing to share substantial personal information but do not like to be surprised by how their data are used [5]. The contextual integrity theory includes the idea that perceptions of privacy are based on ethical concerns that evolve over time [6]. The use of proprietary algorithms to categorize individuals on the basis of behaviors or tendencies can be viewed as ‘creepy.’ In the context of health care, prior work has found that 85% of patients who reported using social media and who were willing to participate in research also agreed to share these data sources and have them linked to their electronic health record for health research [7]. That consent was provided in the context of active patient care, where trust, and also perhaps perceptions of information safeguards, are typically high. Beyond social media however, little is known about what other digital traces patients would willingly share with health researchers, under what circumstances, and for what reason [8].

We used a deception design to credibly evaluate participants’ willingness to share data, the health relatedness of those digital data sources, and preferences associated with data sharing (e.g. desired information to receive in return and the individuals with whom participants were willing to share).

## Methods

### Aim, design and setting

Patients seeking care in a high volume, urban, academic Emergency Department from July to November 2017 were approached by research assistants for study participation. Excluded were patients 1) < 18 years old, 2) non-English speaking, or 3) High acuity and Trauma Level I. Patients were asked to participate in a survey about digital data and informed that consenting participants would be eligible for a 1 in 50 chance of winning a $40 gift card.

### Survey instrument

The 18-question survey (Additional file 1 of the supplementary material), administered to participants on a laptop or tablet, had 5 core components: willingness to donate data for health research (now and at death), health relatedness of digital data, prior experiences with data privacy, data sharing preferences and concerns, and demographic information.

We asked participants about 19 different types of digital data: Facebook, Twitter, Snapchat, Instagram, EMR, genetic data, prescription history, fitness trackers, credit card purchases, tax records, online purchase history, Google searches, music streaming, Yelp reviews, rideshare history, GPS data, email and text message data. These data types were chosen based on a larger project that the Center for Digital Health is conducting.

In an IRB-approved deception design, participants were asked if they would consider donating any of the 19 different data types to health researchers, and were told that if they selected “Yes” that they would be directed to do so immediately, to simulate an actual real time response. Upon finishing this question block, participants were informed that they would not actually be donating their data, and were directed to subsequent survey questions. Participants used a 5-point scale to report how strongly they felt that various types of digital data contained health-related information.

Participants were asked what data they might choose to donate to researchers, what concerns they would have about data donation (e.g. fraud, abuse, misidentification), and who (e.g. friends, family, physician) they would want to have access to their information [9].

### Analysis

Descriptive statistics were used to characterize each of the components of the survey. Exploratory factor analysis (EFA) was conducted to identify clusters of different data sources grouped according to participants’ sense of health-relatedness and willingness to share. EFA was conducted in R 3.5.1 using Parallel analysis [10, 11] comparing the scree of factors of the observed data with that of a random data matrix of the same size.

## Results

Of 595 people approached, 206 (35%) consented to participate. Participants were primarily young, female, African-American, and lower income (Table 1).

**Table 1:** Participant Characteristics

Characteristic	(*n* = 206)
Age	
18–24	32 (16%)
25–34	61 (30%)
35–44	50 (25%)
45–54	23 (11%)
> 55	37 (18%)
Race	
Black	129 (63%)
White	38 (19%)
Hispanic/Latin(o/a)	10 (5%)
Asian/Pacific Islander	2 (1%)
Multiracial	9 (4%)
Other	17 (8%)
Gender	
Female	131 (64%)
Male	71 (35%)
Other	1 (2%)
Education	
< High School	11 (5%)
High School Graduate/GED	81 (40%)
> High School Graduate/GED	113 (55%)
Income	
< $30,000	99 (48%)
$30,000 - $59,999	43 (21%)
> $60,000	30 (15%)
No Answer	33 (16%)

### Willingness to donate digital data for health research

**Fig. 1:**
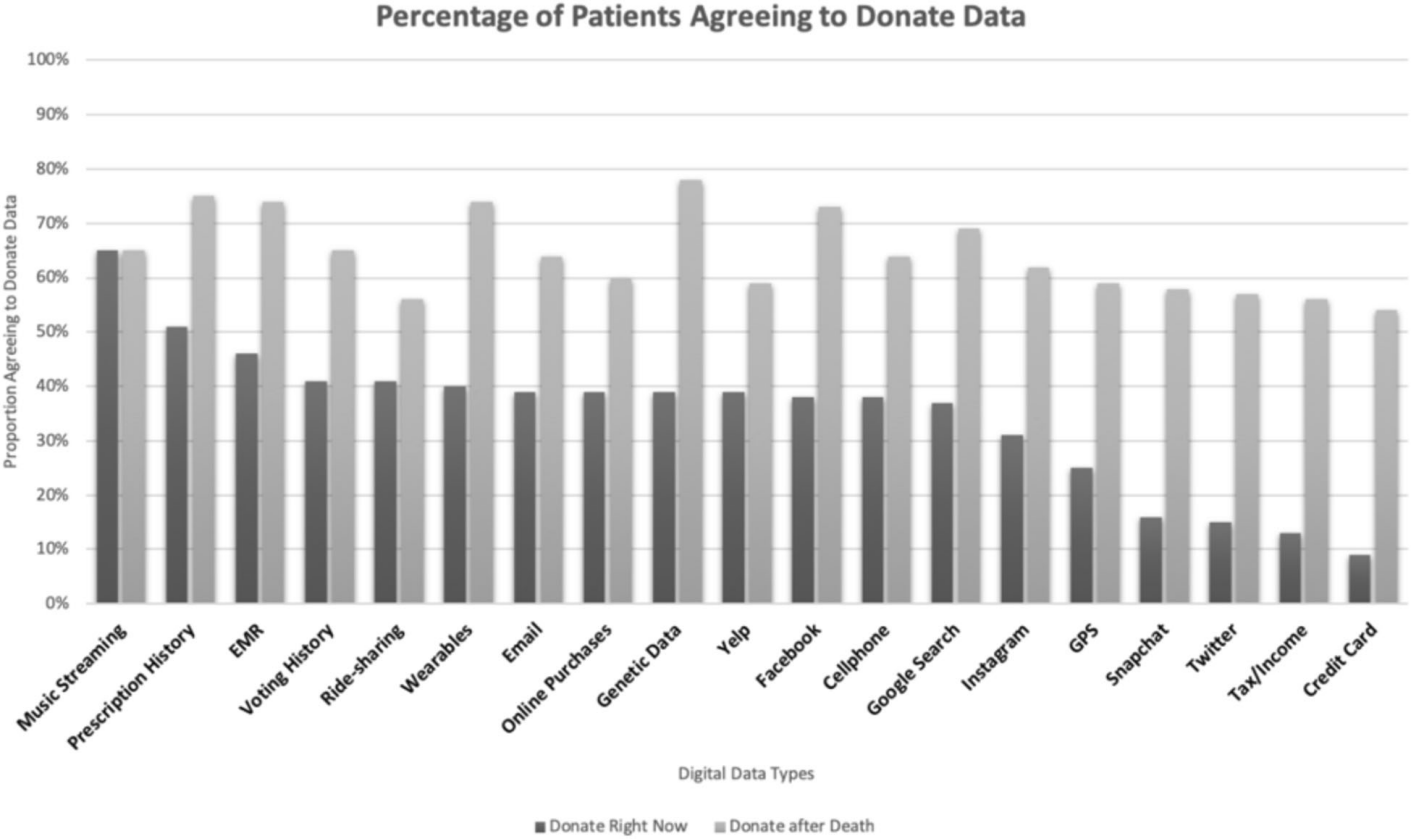
Percentage of Patients Agreeing to Donate Data Now & After Death. This figure indicates the proportion of patients who agreed to donate each data type when approached in the Emergency Department, and the proportion that indicated that they would be willing to donate each data type after their death

Participants’ willingness to share 19 specific types of digital data with researchers at the time of the survey and after death are reported in Fig. 1. One hundred four participants (65%) agreed to share at least one digital data type listed in the survey. Participants were more willing to share digital data after death for all data types.

Factor analysis revealed 6 discrete themes grouping different types of data according to patient willingness to share (Table 2). Based on the dominant content of these data, we interpreted these groupings as Health/location, Social Media, Other activities, Politics, Communication, Financial. Additional file 2: Figure S1 (in the supplement) shows the percentage of patients who reported using the indicated devices or accessing the type of data listed.

**Table 2:** Factor analysis ‘willingness to share’

Data type	Factor loading 1 “Health/Location”	Factor loading 2 “Social Media”	Factor loading 3 “Other activities”	Factor loading 4 “Politics”	Factor loading 5 “Communication”	Factor loading 6 “Financial”
Prescriptions	0.708					
EHR	0.837					
Geolocation	0.641					
Genetic data	0.514					
Facebook		0.339				
Twitter		0.751				
Instagram		0.837				
Snapchat		0.785				
Online purchases			0.345			
Music streaming			0.471			
Yelp			0.659			
Ridesharing			0.763			
Fitness tracker			0.732			
Voting history				0.884		
Email					0.654	
Text message					0.735	
Google search					0.726	
Taxes						0.792
Credit card						0.675

### Health relatedness of digital data

**Fig. 2:**
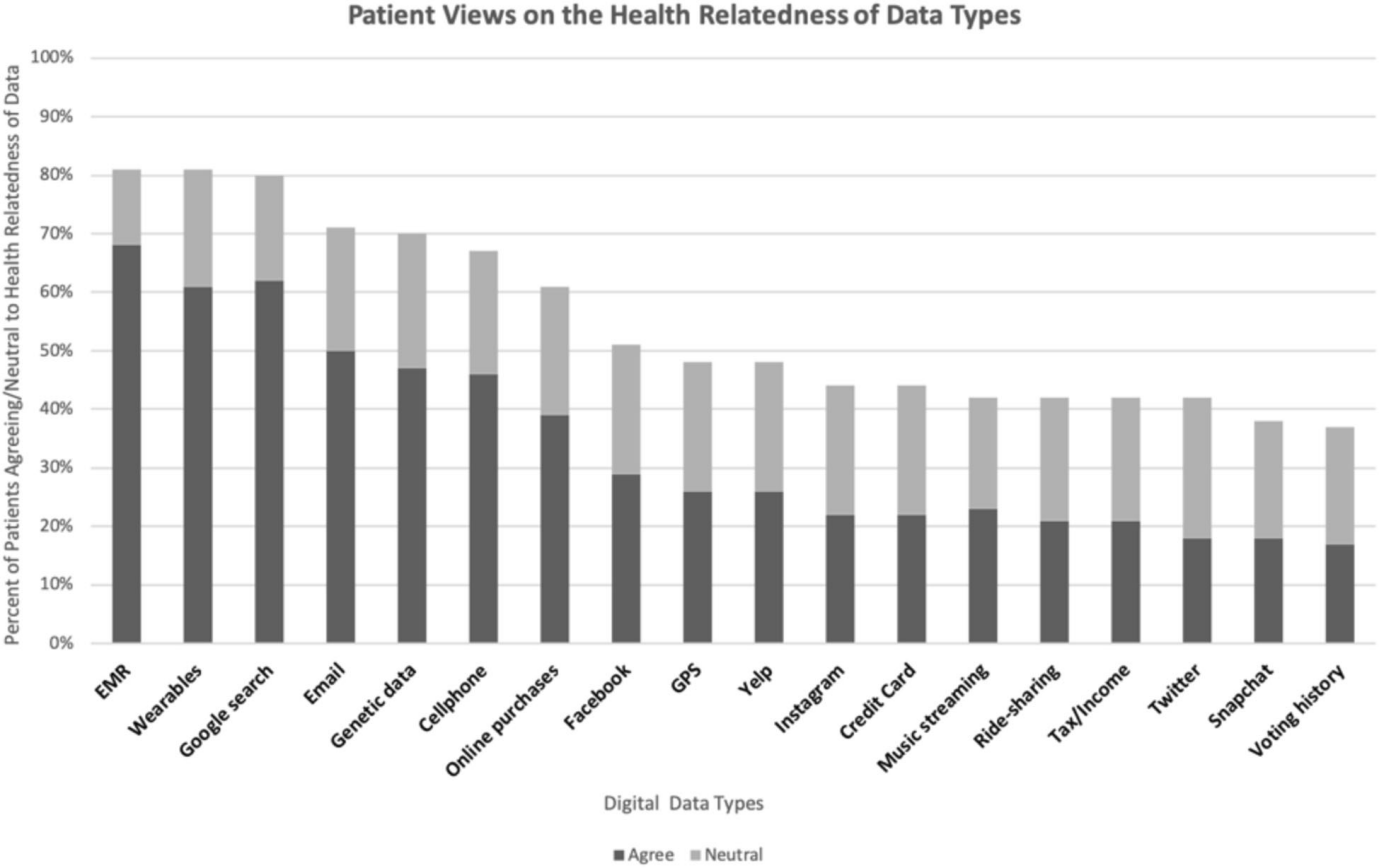
Patient Views on the Health Relatedness of Data Types. This figure represents the proportion of participants who answered either “neutral” or “agree” to whether each of the indicated data types is related to health

Figure 2 reports participants’ assessments of the health relatedness of different data sources. Of note, Google search histories, data from wearables, and email were considered more health related than genetic data.

Factor analysis revealed 5 discrete themes grouping different types of data according to perceived health relatedness (Table 3). Based on the dominant content of these data, we interpreted these groupings as Social Media, Health, Financial/location, Apps, Communication/commerce.

**Table 3:** Factor analysis ‘relatedness to health’

Data type	Factor loading 1 “Social Media”	Factor loading 2 “Heath”	Factor loading 3 “Financial/Location”	Factor loading 4 “Apps”	Factor loading 5 “Communication Commerce”
Facebook	0.634				
Twitter	0.988				
Instagram	0.870				
Snapchat	0.896				
Yelp	0.308				
Google search		0.393			
Genetic data		0.573			
EHR		0.672			
Fitness tracker		0.832			
Taxes			0.613		
Credit card			0.821		
Voting history			0.527		
Geolocation			0.629		
Music streaming				0.844	
Ridesharing				0.463	
Email					0.798
Text message					0.713
Online purchases					0.339

### Data sharing preferences and concerns

Patients were most interested in receiving feedback about potential risk factors 139 (67%) gleaned from their data; 155 patients (72%) wanted the information shared with themselves and 111 (51%) with a health care provider. Only 8 individuals (4%) said they would share health insights with their social network (Table 4).

**Table 4:** Preferences for data sharing and data concerns

Data Preferences	n (%)
Feedback Preferences: If you donated your electronic data to health researchers, what type of feedback would you like to receive?	
Exercise and eating habits	130 (60%)
Habits effect on health	137 (63%)
Language analysis from social media	47 (22%)
Comparison to other donors’ data	62 (29%)
Potential risk factors	140 (65%)
Other	30 (14%)
Result Sharing Preferences: If you donated your electronic data to researchers, who would you want insights from your data to be shared with?	
Myself	155 (72%)
Researchers	77 (36%)
Health care provider	111 (51%)
Social network	8 (4%)
Family	89 (41%)
Others with similar health conditions	58 (27%)
General Privacy Preferences: I am generally a private person in my everyday life.	
Agree	175 (85%)
Disagree	22 (11%)
Unsure	9 (4%)
I tend to reveal minimal personal information about myself online due to privacy concerns.	
Agree	157 (76%)
Disagree	33 (16%)
Unsure	16 (8%)
I feel uncomfortable when other people have access to my personal information.	
Agree	157 (76%)
Disagree	27 (13%)
Unsure	22 (11%)
I believe that there is no need to be concerned about revealing personal information online.	
Agree	44 (21%)
Disagree	149 (72%)
Unsure	13 (6%)
It does not bother me that a history of my online activities may be available to 3rd parties online.	
Agree	41 (20%)
Disagree	146 (71%)
Unsure	19 (9%)
I regularly use anti-virus/phishing/spamming software, or clear my browser history/cookies/cache.	
Agree	102 (50%)
Disagree	78 (38%)
Unsure	26 (13%)
Digital Data Concerns (Yes vs. No): Information I share with friends online may be inappropriately disclosed by them to others.	
Yes	115 (56%)
No	66 (32%)
Unsure	25 (12%)
People who you only know from online are not who they say they are.	
Yes	109 (53%)
No	54 (26%)
Unsure	43 (21%)
Other internet users might try to defraud you or abuse your personal information.	
Yes	150 (73%)
No	31 (15%)
Unsure	25 (12%)
Online companies and websites might try and share your information to other parties without explicit consent.	
Yes	153 (74%)
No	34 (17%)
Unsure	19 (9%)
Online companies and websites might use your information for purposes not explicitly stated in the privacy policy.	
Yes	149 (72%)
No	34 (17%)
Unsure	23 (11%)

Patients also expressed concerns about potential data and privacy breaches; a majority were concerned that friends online might inappropriately disclose private information to others 115 (56%), that they might be defrauded online or their personal information would be abused 149 (73%), that companies might share information with third parties without consent 153 (74%), and that companies and websites might use their information in ways not stated in the privacy policies or user agreements 149 (72%).

## Discussion

This study has three main findings. First, patients in this study were willing to share several non-traditional forms of data with health researchers now and even more so after they have died. Second, a non-trivial percentage of patients recognized that digital footprints left in non-health areas such as finance or commerce may reveal information about their health. Third, these patients have preferences about what health related insights they would want to learn from their digital data and with whom they would want to share this information, and potential pitfalls of digital data sharing.

Participants were willing to share many types of digital data with researchers, some revealing a willingness to share presumably sensitive data like tax records and credit card purchases. These financial data sources may be highly predictive of health and health outcomes [12]. There are many steps however between sharing and actionable information [13, 14]. Each data source provides different signal, and the extent of the potential signal is likely mediated by the amount of data shared, and how individualized that data are. A growing literature addresses correlations between digital data and health outcomes and health care utilization [15–22]. Much of this research relies on participants sharing personal data with researchers. Less is known however about patients’ perceptions about how connected these data are with their health.

The connection between many of these data sources to health is often obvious and many of those health connections were frequently recognized by study participants. And yet, regulations protecting the privacy of health information are defined not by health-relatedness, but by information source [23]. Health-related information arising in the context of clinical care is highly protected under the Health Insurance Portability and Accountability Act. Health-related information arising in the context of consumer purchases or social media use is not. And yet in some cases that latter was perceived as more health related than the former.

The emergence of direct-to-consumer genetic testing sites like 23andMe [24–26] can reveal predictive or suggestive information to patients that they may or may not want to know. For example, when considering feedback from genetic research, 87% of participants agree that they would want to have findings shared with them if researchers found that they had a genetic pattern linked to a life threatening condition, which was manageable or curable, 73% if the condition was not life threatening, and 72% if the condition was life threatening but not curable [27]. We found similar percentages when we asked survey respondents if they would want to know if patterns in their digital data indicate that they had a higher than average risk for a treatable disease (85%). When asked if patterns in their data indicated that they had a higher than average risk for a non-treatable disease 75% would want to know, and if patterns in their data indicated that they had a lower than average risk 74% would want to know.

In April 2018, it was revealed that the firm Cambridge Analytica had accessed the data of more than 80 million individuals’ Facebook accounts without their permission. The public response was considerable, with many concerns raised about data privacy and what companies know about individuals and the types of information they share online. This type of large-scale privacy violation has an impact on the trust people have in the security of their digital data, and some people reportedly deleted their social media accounts after this occurred [28]. Of note, a large proportion (74%) of patients surveyed in this study had expressed concern that companies might share information with third parties without consent, a full 6-months before the Cambridge Analytica activities were reported in the media.

As researchers gain greater insight into the relationship between online activity and an individual’s health, transparency of these findings is essential to maintain trust. Increasing focus on returning research findings to patients is evident in the digital era where there is a movement toward open science and better patient engagement [29].

A better understanding specifically of health-related digital footprints is important for being able to provide guidance to patients about their use of digital platforms and sharing practices. This emerging field is in its infancy as many of the most popular social media and online sites have only been available for slightly more than ten years.

While providing data back to patients would be a first step, future work would also focus on the utility of this data being provided to healthcare providers via an EMR. Less defined is how this data would be interpreted, or used, or if it would even be welcomed. Regular reports of patients’ steps walked, calories consumed, Facebook status updates, and online footprints might create overwhelming expectations of regular surveillance of questionable value and frustratingly limited opportunities to intervene even if strong signals of abnormal patterns were detected [30]. This future work could assess healthcare providers use of digital data incorporated in an EMR and focus on issues related to the accuracy, interpretability, meaning, and actionability of the data [31–35].

This study has limitations. The findings are exploratory and represent a small sample size from a non-representative population. Response rate may have been influenced by patients being queried in a medical environment and could vary if patients were asked in non-hospital settings. This study also has strengths. Because we told patients that we would immediately access their data should they be willing to share it, their willingness to share more likely represents true preferences, rather than merely the expressed preferences of a typical hypothetical setting.

## Conclusions

Patients use a variety of digital applications that generate large amounts of data. Our work demonstrates that participants would be willing to donate some of their digital data to researchers and clinicians in pursuit of health-related insights. This work adds to the larger domain of privacy and health research by connecting various digital data with perceived health relatedness. Both the willingness to share data and the perceived relatedness of those data to health do not follow conventional divisions on which health information privacy policies are built. Future work should be directed towards understanding the contexts in which patients are most likely to donate data for research use, and how they would want insights shared with them.

## Additional files

Additional file 1: Survey Questionnaire. (DOCX 35 kb)

Additional file 2: **Figure S1.** Patients Reporting Data Usage/Access. This figure shows the percentage of patients who reported using the indicated devices or accessing the type of data listed. (DOCX 74 kb)

## Data Availability

The datasets generated and/or analyzed during the current study are not publicly available due to the IRB guidelines but are available from the corresponding author on reasonable request.

## Abbreviations

EFA: Exploratory factor analysis
EMR: Electronic Medical Records
GPS: Global Position System
HIPAA: Health Insurance Portability and Accountability Act of 1996
IRB: Institutional Review Board
