# Supplementary material for: Patients’ willingness to share digital health and non-health data for research: a cross-sectional study
Source: BMC Med Inform Decis Mak. 2019 Aug 8;19:157. doi: 10.1186/s12911-019-0886-9 (PMC6686530; doi:10.1186/s12911-019-0886-9)
Supplement: Supplementary file 1 — Survey Questionnaire. (DOCX 35 kb) [file 12911_2019_886_MOESM1_ESM.docx]

Survey Questionnaire

Start of Block: Welcome

Welcome **Welcome to the Data Preferences and Privacy Study, a project of the University of Pennsylvania Center for Digital Health!**

 Our goal is to determine how Penn Medicine patients think and feel about many different types of electronic data, and the security and privacy of that data. We also want to learn how Penn Medicine patients feel about donating different types of data to health research, now or in the future.   
 
Thank you for participating, and as a reminder you can choose not to continue at any time. 

End of Block: Welcome

Start of Block: Willingness to Share

| 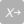 |
| --- |

DSHAREWILLING Are you willing to share the following types of data with researchers today? If yes is selected, you will be asked to provide us with access to this data next.

|  | Yes (1) | No (0) | I don't use this source of data (88) |
| --- | --- | --- | --- |
| Facebook data (DSHAREWILLING_1) |  |  |  |
| Twitter data (DSHAREWILLING_15) |  |  |  |
| Instagram data (DSHAREWILLING_16) |  |  |  |
| Snapchat data (DSHAREWILLING_17) |  |  |  |
| Email History (Gmail, Yahoo, Comcast, Verizon, etc.) (DSHAREWILLING_2) |  |  |  |
| Text Message and Phone Call Data (DSHAREWILLING_3) |  |  |  |
| Google search history (DSHAREWILLING_4) |  |  |  |
| Online purchase history (Amazon, Target, Ebay, etc.) (DSHAREWILLING_5) |  |  |  |
| Music streaming data (Spotify, Pandora, etc.) (DSHAREWILLING_14) |  |  |  |
| Yelp reviews and ratings (DSHAREWILLING_6) |  |  |  |
| Ride-sharing history (Uber, Lyft, etc.) (DSHAREWILLING_7) |  |  |  |
| Fitness tracker data (FitBit, Apple Watch, etc.) (DSHAREWILLING_8) |  |  |  |
| Tax records and income history (DSHAREWILLING_9) |  |  |  |
| Credit card statement data (DSHAREWILLING_10) |  |  |  |
| Voting history (DSHAREWILLING_11) |  |  |  |
| Prescription history (CVS, Walgreen's, etc.) (DSHAREWILLING_12) |  |  |  |
| Electronic medical record data (DSHAREWILLING_18) |  |  |  |
| Geolocation (GPS from your phone or computer) data (DSHAREWILLING_13) |  |  |  |
| Genetic data (23andMe) (DSHAREWILLING_20) |  |  |  |

End of Block: Willingness to Share

Start of Block: Debrief

WHYSHARE Thank you for answering the above questions about your willingness to share certain types of data with us. We will not be collecting any of your data, but we would like you to answer some more questions about your responses.

End of Block: Debrief

Start of Block: Data Health Relationship

| 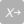 |
| --- |

HR_ALL The following questions will ask you about the types of data as the question before, but instead of answering about willingness to share, we want to find out if you think each type of data is related to health.
 
For each data type, please click how much you feel it contains health related information, from "Strongly Agree" to "Strongly Disagree." 
 
For example, "Google search data" might be used to see if a patient with diabetes is searching for a new treatment or a blood glucose monitor.  Another example might be if researchers collect social media posts, and look at language for early signs of memory loss.

|  | Strongly Agree (5) | Agree (4) | Neutral (3) | Disagree (2) | Strongly Disagree (1) |
| --- | --- | --- | --- | --- | --- |
| **Facebook data** contain health-related information. (HR_ALL_1) |  |  |  |  |  |
| **Twitter data** contain health-related information. (HR_ALL_2) |  |  |  |  |  |
| **Instagram data** contain health-related information. (HR_ALL_3) |  |  |  |  |  |
| **Snapchat data** contain health-related information. (HR_ALL_4) |  |  |  |  |  |
| **Email accounts** (Gmail, Yahoo, Comcast, Verizon, etc.) contain health-related information. (HR_ALL_5) |  |  |  |  |  |
| **Text message data and phone call history** contains health-related information (HR_ALL_6) |  |  |  |  |  |
| **Google search history** contains health-related information. (HR_ALL_7) |  |  |  |  |  |
| **Online purchase history** (Amazon, Target, Google Buy, Ebay, etc.) contains health-related information. (HR_ALL_8) |  |  |  |  |  |
| **Music streaming data** (Spotify, Pandora, Apple Music, etc.) contains health-related information. (HR_ALL_9) |  |  |  |  |  |
| **Electronic Medical Records** contain health-related information. (HR_ALL_38) |  |  |  |  |  |
| **Yelp reviews and ratings** contain health-related information. (HR_ALL_10) |  |  |  |  |  |
| **Ride-sharing history** (Uber, Lyft, etc.) contains health-related information. (HR_ALL_11) |  |  |  |  |  |
| **Fitness Tracker/Wearables history** contains health-related information. (HR_ALL_12) |  |  |  |  |  |
| **Tax records and other income history** contains health-related information. (HR_ALL_13) |  |  |  |  |  |
| **Credit card statements** contain health-related information. (HR_ALL_14) |  |  |  |  |  |
| **Voting history** contains health-related information. (HR_ALL_15) |  |  |  |  |  |
| **Geolocation** (GPS from your phone or computer) data contains health-related information. (HR_ALL_18) |  |  |  |  |  |
| **Genetic data** (23andMe) contains health-related information (HR_ALL_37) |  |  |  |  |  |

End of Block: Data Health Relationship

Start of Block: Expectations

DFEEDBACK If you donated your electronic data to health researchers, what type of feedback would you like to receive? (Check all that apply)

- Information about my eating and exercise habits (1)
- Information about how my habits contribute to my health (2)
- Health-related language analysis of my social media posts (3)
- Comparison my health data to other donors' data (4)
- Potential risk factors for health conditions (5)
- Other (6)

Display This Question:

If If you donated your electronic data to health researchers, what type of feedback would you like t... = Other

DFEEDBACK_OTHER What other types of feedback would you like to receive from researchers?

________________________________________________________________

| 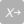 |
| --- |

KNOWRISK For each of the following scenarios, please indicate whether or not you would want to know the results of an analysis of your donated data.

|  | I would want to know (1) | I wouldn't want to know (0) | Unsure (98) |
| --- | --- | --- | --- |
| Patterns in your data indicate that you are at higher-than-average risk for a treatable disease, like high blood pressure or asthma. (2) |  |  |  |
| Patterns in your data indicate that you are at higher-than-average risk for a non-treatable disease, like Alzheimers disease. (3) |  |  |  |
| Patterns in your data indicate that you are at lower-than-average risk for any type of health condition. (4) |  |  |  |

| 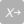 |
| --- |

OTHBENEFIT Do you think others could benefit from you donating your electronic data?

- Yes (1)
- Maybe (2)
- No (0)

DATACONCERN Do you have any concerns about donating your electronic data to researchers in the future?

________________________________________________________________

________________________________________________________________

________________________________________________________________

________________________________________________________________

________________________________________________________________

SHRDATA If you donated your electronic data to researchers, who would you want insights from your data to be shared with? (Check any that apply)

- Myself (1)
- Family (6)
- Researchers (2)
- Doctor/healthcare provider (3)
- Social network (4)
- Others with health conditions like mine (5)

| 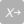 |
| --- |

DONATEDEATH If the opportunity arose in the future, would you consider donating/willing your electronic data to health researchers after death?

- Yes, all of my data (2)
- Yes, but only some of my data (1)
- No (0)
- Unsure (98)

Display This Question:

If If the opportunity arose in the future, would you consider donating/willing your electronic data... = Yes, but only some of my data

DEATHDATATYPE Which types of data would you feel comfortable donating after death? (Check all that apply)

- Facebook data (4)
- Twitter data (5)
- Instagram data (6)
- Snapchat data (7)
- Email History (Gmail, Yahoo, Comcast, Verizon, etc.) (8)
- Text Message and Phone Call Data (9)
- Google search history (10)
- Online purchase history (Amazon, Target, Ebay, etc.) (11)
- Music streaming data (Spotify, Pandora, etc.) (12)
- Yelp reviews and ratings (13)
- Ride-sharing history (Uber, Lyft, etc.) (14)
- Fitness tracker data (FitBit, Apple Watch, etc.) (15)
- Tax records and income history (16)
- Credit card statement data (17)
- Voting history (18)
- Prescription history (19)
- Electronic medical record data (20)
- Geolocation (GPS from your phone or computer) data (21)
- Genetic data (22)

End of Block: Expectations

Start of Block: Privacy Threshold

Opinion This section will ask you questions about your opinions and beliefs about various types of online activities.

| 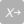 |
| --- |

TRUST Are you **concerned** with the following statements?

|  | Yes (1) | No (0) | Unsure (98) |
| --- | --- | --- | --- |
| Information I share with friends online may be inappropriately disclosed by them to others. (TRUST_1) |  |  |  |
| People who you only know from online are not who they say they are. (TRUST_2) |  |  |  |
| Other internet users might try to defraud you or abuse your personal information. (TRUST_3) |  |  |  |
| Online companies and websites might try and share your information to other parties without explicit consent. (TRUST_4) |  |  |  |
| Online companies and websites might use your information for purposes not explicitly stated in the privacy policy. (TRUST_5) |  |  |  |

| 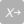 |
| --- |

PRIVACY Do you **agree or disagree** with the following statements?

|  | Agree (1) | Disagree (0) | Unsure (98) |
| --- | --- | --- | --- |
| I am generally a private person in my everyday life. (PRIVACY_1) |  |  |  |
| I tend to reveal minimal personal information about myself online due to privacy concerns. (PRIVACY_2) |  |  |  |
| I feel uncomfortable when other people have access to my personal information. (PRIVACY_3) |  |  |  |
| I believe that there is no need to be concerned about revealing personal information online. (PRIVACY_4) |  |  |  |
| It does not bother me that a history of my online activities may be available to 3rd parties online. (PRIVACY_5) |  |  |  |
| I regularly use anti-virus/phishing/spamming software, or clear my browsing history/cookies/cache. (PRIVACY_6) |  |  |  |

| 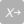 |
| --- |

ADVERSE Have any of the following events happened to you online?

|  | Yes (1) | No (0) | Unsure (98) |
| --- | --- | --- | --- |
| An account of yours was accessed by someone you didn't give permission. (ADVERSE_1) |  |  |  |
| The privacy of your personal information was violated. (ADVERSE_2) |  |  |  |
| Your reputation was negatively affected as a result of information posted online. (ADVERSE_3) |  |  |  |
| You had an unpleasant experience as a result of information you gave out online. (ADVERSE_4) |  |  |  |
| You were the victim of fraud and/or identity theft. (ADVERSE_5) |  |  |  |

End of Block: Privacy Threshold

Start of Block: Demographics

| 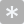 |
| --- |

AGE What is your date of birth? (MM/DD/YYYY format)

________________________________________________________________

| 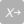 |
| --- |

RACE With which race/ethnicity do you identify? (Choose one)

- Black/African American (1)
- White (2)
- Hispanic/Latino(a) (3)
- Asian/Pacific Islander (4)
- Native American (5)
- Multiracial (6)
- Other (88) ________________________________________________

| 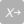 |
| --- |

GENDER With which gender do you most identify?

- Female (1)
- Male (2)
- Male-to-Female Transgender (3)
- Female-to-Male Transgender (4)
- Intersex (6)
- Other (88) ________________________________________________

| 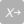 |
| --- |

INCOME Within what range does your average annual income fall?

▼ 0 - $9,999 (0) ... Refuse to Answer (99)

| 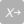 |
| --- |

EDUCATION What is the highest level of education that you have completed?

- Less than high school (1)
- High school graduate/GED (2)
- Some college (3)
- 2 year degree (4)
- 4 year degree (5)
- Professional degree (6)
- Doctorate (7)

End of Block: Demographics
