# Supplementary material for: Patients’ willingness to share digital health and non-health data for research: a cross-sectional study
Source: BMC Med Inform Decis Mak. 2019 Aug 8;19:157. doi: 10.1186/s12911-019-0886-9 (PMC6686530; doi:10.1186/s12911-019-0886-9)

**Figure S1: Patients Reporting Data Usage/Access**

This figure shows the percentage of patients who reported using the indicated devices or accessing the type of data listed.

Figure S1: Patients Reporting Data Usage/Access


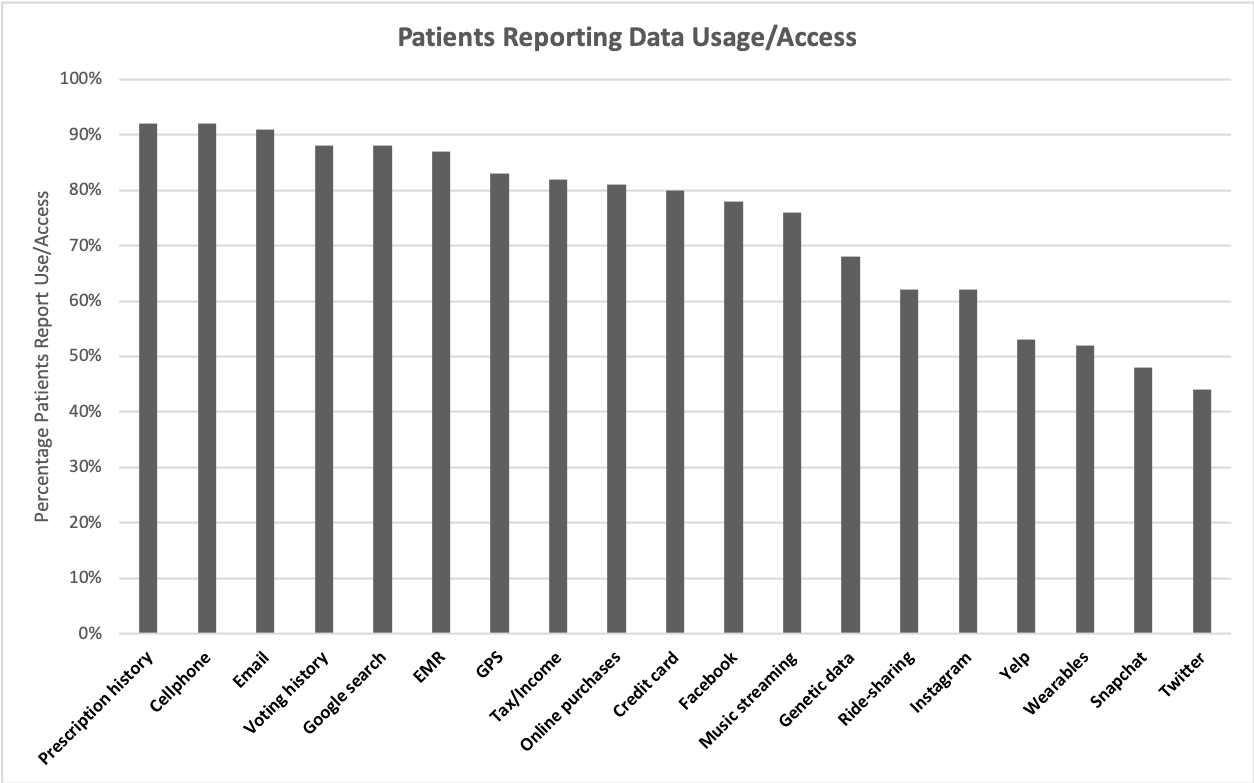

Supplement: Supplementary file 2 — Figure S1. Patients Reporting Data Usage/Access. This figure shows the percentage of patients who reported using the indicated devices or accessing the type of data listed. (DOCX 74 kb) [file 12911_2019_886_MOESM2_ESM.docx]
